# Modal Performance of Two-Fiber Orthogonal Gradient Composite Laminates Embedded with SMA

**DOI:** 10.3390/ma13051102

**Published:** 2020-03-02

**Authors:** Yizhe Huang, Zhifu Zhang, Chaopeng Li, Kuanmin Mao, Qibai Huang

**Affiliations:** State Key Laboratory of Digital Manufacturing Equipment and Technology, Huazhong University of Science and Technology, Wuhan 430074, China; yizhehuang@foxmail.com (Y.H.); jeff.zfzhang@foxmail.com (Z.Z.); 15927673439@163.com (C.L.); maokm@hust.edu.cn (K.M.)

**Keywords:** shape memory alloys, two-phase fiber, gradient laminate, modal performance

## Abstract

A gradient composite laminate that was composed of two-phase fibers, a shape memory alloy (SMA), and graphite was prepared to investigate modal performance and improve vibration behavior. The stress-strain relation of the single-layer composite plates was derived from Kirchhoff thin plate theory and the material constitutive of the SMA. A gradient distribution model and the eigenvalue equations of gradient composite laminates were developed. The influence of the fiber component content gradient distribution, pre-strain, the two-phase fiber volume fraction, and geometric parameters on the modal performance was analyzed. This study provides a method to avoid the structural resonance of composite laminates that are embedded with an SMA through the gradient distribution of two-phase fiber content that leads to the interaction of the material properties.

## 1. Introduction

The natural frequency of traditional fiber-reinforced composite laminates is determined under certain structural size and boundary conditions that directly affect vibration and sound radiation. When the external excitation frequency is the same as or close to the natural frequency of the composite laminate structure, there will be resonance in the composite laminates, and the noise will be larger. In contrast, by adding a shape memory alloy (SMA) and graphite-embedded material to the substrate, the natural frequency can be adjusted without modifying the original shape dimensions and boundary conditions of the laminates. Such modification avoids structural resonance and reduces structural vibration and noise.

An SMA has high temperature sensitivity. The modulus of elasticity and Poisson’s ratio of the SMA changes versus temperature. The recovery stress of the pre-strain also varies with temperature. Since the temperature effect can break through the limitations of the traditional laminates due to material characteristics and original shape, research on the vibration and buckling of composite laminates that are embedded within SMAs has aroused the research interest of many scholars. Rogers et al. [1,2,3] used the Rayleigh–Ritz method to examine the linear statics and dynamics of composite laminates with SMA fibers, and they performed experiments on active vibration and structural acoustics. Ostachowicz et al. [4] demonstrated the effect of nitinol fiber on the natural frequency analysis of composite materials, and they established a finite element model for predicting the natural frequency and modes of vibration. Malekzadeh et al. [5,6] investigated the dynamic behavior of a composite plate based on detailed parametric analyses such as those of the volume fraction, pre-strain, orientation, the location of the SMA, and the aspect ratio of the plate. When the thermal stresses increase to a certain critical point, the structure may lose its elastic stability, thus leading to thermal buckling. Therefore, several studies have used refined theory and finite element methods to investigate the vibration of thermally buckling and post-buckled composite plates that are reinforced with SMAs [7,8,9,10,11,12,13,14,15].

In recent years, the integration of SMAs into functionally graded materials (FGMs) has emerged as a new research hotspot [16,17,18,19,20]. Birman [21] proposed the advantage of the sinusoidal distribution of SMA fibers that results in a significant increase in the buckling load. Sepiani et al. [22] found that changes in the thermally driven behavior of a shape memory alloy (SMA)/FGM actuator’s responses during phase transformation due to strain recovery are significant. Asadi et al. [23] and Babaee et al. [24] both studied the nonlinear thermal buckling of an SMA layer and a functionally graded beam/plate on nonlinear elastic foundation. The type of SMA/FG layer has a large influence on the nonlinear buckling solution, which can significantly delay the thermal bending temperature. Furthermore, the induced tensile recovery stress of SMA fibers can also stabilize geometrically imperfect beams/plates. However, few studies to date have focused on improving vibration behavior by investigating the gradient distribution of SMAs and graphite as reinforcements. For this study, a dynamic model of composite laminates with an SMA gradient distribution was developed. The influence of nitinol/graphite fibers on the free vibration behavior of orthogonal laminates was investigated by varying the gradient distribution, pre-strain, fiber volume fraction, and structural size parameters with temperature. The relationship between the gradient distribution of two-phase fibers and natural frequencies provides a technical basis for the vibration control of composite plate structures.

## 2. Formulation

**Figure 1:**
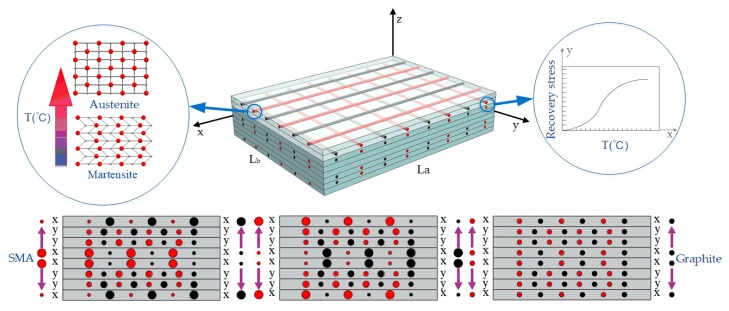
Schematic of an orthogonal gradient laminate that is reinforced by a two-phase fiber with nitinol/graphite.

Consider a rectangular SMA-embedded composite laminate with length *L_a_*, width *L_b_*, and thickness *h* (Figure 1). The nitinol/graphite fiber is embedded in the orthogonal directions. The direction of the two fibers is consistent for a certain layer, i.e., the longitudinal direction or the transverse direction. Two-phase fibers are symmetrically buried on both sides of the mid-plane of the laminates. SMA fibers are stretched to a certain plastic deformation before embedding. During the curing process of composites at high temperature, the ends of the fibers are fixed to prevent the fibers from shrinking to their ‘memory’ length. Thus, the SMA fibers with plastic deformation form a whole with the composites or structures. When the SMA is activated by heating the fibers (through electrification), the shape memory fibers shrink and try to restore their original shape. The shear force along the entire length of the fibers is distributed. At the same time, the Young’s modulus of the SMA fibers changes accordingly. These two factors can be used to control the bending stiffness of the structure and vary its modal performance.

The displacement field may be expressed as:

(1) $$u\left(x,y,z,t\right)=u_{0}\left(x,y,t\right)-z\frac{\partial w\left(x,y,t\right)}{\partial x}$$

(2) $$v\left(x,y,z,t\right)=v_{0}\left(x,y,t\right)-z\frac{\partial w\left(x,y,t\right)}{\partial y}$$

(3) w(x,y,z,t)=w(x,y,t)

where $u_{0}\left(x,y,t\right)$ and $v_{0}\left(x,y,t\right)$ are the in-plane displacement components in the mid-plane of the plate and w(x,y,t) is the out-of-plane displacement components in the mid-plane of the plate. The kinematic relation can be determined as:

(4) $$\left(\begin{array}{c} \varepsilon_{x}\\ \varepsilon_{y}\\ \gamma_{xy} \end{array}\right)=\left(\begin{array}{c} \frac{\partial u_{0}}{\partial x}\\ \frac{\partial v_{0}}{\partial y}\\ \frac{\partial u_{0}}{\partial y}+\frac{\partial v_{0}}{\partial x} \end{array}\right)+z\left(\begin{array}{c} -\frac{\partial^{2}w}{\partial x^{2}}\\ -\frac{\partial^{2}w}{\partial y^{2}}\\ -2\frac{\partial^{2}w}{\partial x\partial y} \end{array}\right)=\left(\varepsilon_{0}\right)+z\left(\Gamma \right)$$

where $\varepsilon^{0}$ and Γ are the mid-plane strain and plate curvature, respectively.

Composite laminates are laminated by an epoxy resin-embedded, nitinol/graphite-reinforced material. The substrate that is composed of graphite/epoxy envelops the SMA fibers therein to make the SMA-reinforced lamina. Each composite layer has an orthogonal fiber orientation. The laminate contains *n* layers, and the volume of composite laminates can be expressed as $V=L_{a}\times L_{b}\times h$. The thickness *h* can equivalently represent the volume of the composite laminates with a certain length and width. When the volume percentage of the SMA in composite laminates is *Vs*%, the volume of the SMA can be expressed as *h* × *Vs*%. In symmetric composite laminates in which the SMA fibers are uniformly distributed, the SMA fiber volume of each layer is $\left(h/n\right)\times V_{s}\%$. Considering the symmetry of composite laminates, the SMA fiber volume of half (i.e., *h*/2) of the laminates is $\left(n/2\right)\times \left[\left(h/n\right)\times V_{s}\%\right]$. The volume fraction of the SMA in each layer of the composite laminates is shown in Equations (5) and (6), while the total content of the SMA fibers in the laminates remains unchanged.

(5) $${\left(\frac{2z_{1}}{h}\right)}^{p_{+}}a_{p+}\left(\frac{h}{n}\times V_{s}\%\right)+{\left(\frac{2z_{2}}{h}\right)}^{p_{+}}a_{p+}\left(\frac{h}{n}\times V_{s}\%\right)+\ldots +{\left(\frac{2z_{n/2}}{h}\right)}^{p_{+}}a_{p+}\left(\frac{h}{n}\times V_{s}\%\right)=\frac{n}{2}\left(\frac{h}{n}\times V_{s}\%\right)$$

(6) $${\left(\frac{h}{2z_{n/2}}\right)}^{p_{-}}a_{p-}\left(\frac{h}{n}\times V_{s}\%\right)+{\left(\frac{h}{2z_{n/2-1}}\right)}^{p_{-}}a_{p-}\left(\frac{h}{n}\times V_{s}\%\right)+\ldots +{\left(\frac{h}{2z_{1}}\right)}^{p_{-}}a_{p-}\left(\frac{h}{n}\times V_{s}\%\right)=\frac{n}{2}\left(\frac{h}{n}\times V_{s}\%\right)$$

The physical parameters p and distribution coefficient a are as follows:

(7) $$P=\left\{p_{+},p_{0},p_{-}\right\}$$

(8) $$a_{p+}=\frac{n/2}{{\left(\frac{2z_{1}}{h}\right)}^{p_{+}}+{\left(\frac{2z_{2}}{h}\right)}^{p_{+}}+\cdots +{\left(\frac{2z_{n/2}}{h}\right)}^{p_{+}}}$$

(9) $$a_{p-}=\frac{n/2}{{\left(\frac{h}{2z_{n/2}}\right)}^{p_{-}}+{\left(\frac{h}{2z_{n/2-1}}\right)}^{p_{-}}+\cdots +{\left(\frac{h}{2z_{1}}\right)}^{p_{-}}}$$

Thus, the volume fraction of the k-th layer in a laminate can be expressed as:

(10) $$V_{sp+}\left(k\right)=\{\begin{array}{c} {\left(\frac{2\left[z_{n/2}-\left(k-1\right)z_{1}\right]}{h}\right)}^{p_{+}}a_{p+}V_{s}\%\;k=1,2,\ldots ,\frac{n}{2}\;\\ {\left[\frac{2\left(k-\frac{n}{2}\right)z_{1}}{h}\right]}^{p_{+}}a_{p+}V_{s}\%\;k=\frac{n}{2}+1,\frac{n}{2}+2,\ldots ,n \end{array}$$

(11) $$V_{sp-}\left(k\right)=\{\begin{array}{c} {\left(\frac{h}{2kz_{1}}\right)}^{p_{-}}a_{p-}V_{s}\%\;k=1,2,\ldots ,\frac{n}{2}\;\\ {\left[\frac{h}{2\left[z_{n/2}-\left(k-1-\frac{n}{2}\right)z_{1}\right]}\right]}^{p_{-}}a_{p-}V_{s}\%\;k=\frac{n}{2}+1,\frac{n}{2}+2,\ldots ,n \end{array}$$

As a physical parameter, the gradient distribution of the SMA fiber content in gradient laminates is determined by the value of *P*.

The stress–strain relation for the orthotropic nitinol/graphite/epoxy ply can be expressed by:

(12) $$\sigma^{\left(k\right)}={\tilde{Q}}^{\left(k\right)}\varepsilon^{\left(k\right)}-{\overset{¯}{Q}}^{\left(k\right)}\alpha^{\left(k\right)}\Delta T+\sigma_{r}V_{s}{}^{\left(k\right)}$$

where ${\tilde{Q}}^{\left(k\right)}$ is the transformed reduced stiffness of the *k*-th ply of the SMA composite laminate and ${\overset{¯}{Q}}^{\left(k\right)}$ is the transformed reduced stiffness of the *k*th ply of the substrate [4,5]. The term $\sigma_{r}$ denotes the temperature-dependent SMA recovery stress in the SMA fiber direction. The second and third terms on the right of the formula represent the thermal stress that is generated by the matrix and the recovery stress that is generated by the SMA fiber, respectively.

For the nitinol/graphite fibers that are distributed in the x direction, the stress–strain relation of the *k*-th composite plate can be expressed as:

(13) $${\left(\begin{array}{c} \sigma_{x}\\ \sigma_{y}\\ \tau_{xy} \end{array}\right)}_{11}^{\left(k\right)}={\left(\begin{array}{ccc} \frac{R_{11}}{1-\mu_{12}\mu_{21}} & \frac{R_{22}\mu_{12}}{1-\mu_{12}\mu_{21}} & 0\\ \frac{R_{22}\mu_{12}}{1-\mu_{12}\mu_{21}} & \frac{R_{22}}{1-\mu_{12}\mu_{21}} & 0\\ 0 & 0 & H_{12} \end{array}\right)}^{\left(k\right)}{\left(\begin{array}{c} \varepsilon_{x}\\ \varepsilon_{y}\\ \gamma_{xy} \end{array}\right)}^{\left(k\right)}-{\left(\begin{array}{ccc} \frac{E_{11}}{1-\nu_{12}\nu_{21}} & \frac{E_{22}\nu_{12}}{1-\nu_{12}\nu_{21}} & 0\\ \frac{E_{22}\nu_{12}}{1-\nu_{12}\nu_{21}} & \frac{E_{22}}{1-\nu_{12}\nu_{21}} & 0\\ 0 & 0 & G_{12} \end{array}\right)}^{\left(k\right)}{\left(\begin{array}{c} \alpha_{11}\\ \alpha_{22}\\ 0 \end{array}\right)}^{\left(k\right)}\Delta T+{\left(\begin{array}{c} \sigma_{r}V_{s}\\ 0\\ 0 \end{array}\right)}^{\left(k\right)}$$

Similarly, for the nitinol/graphite fibers that are distributed in the y direction, the stress–strain relation of the *k*-th layer can be expressed as:

(14) $${\left(\begin{array}{c} \sigma_{x}\\ \sigma_{y}\\ \tau_{xy} \end{array}\right)}_{22}^{\left(k\right)}={\left(\begin{array}{ccc} \frac{R_{22}}{1-\mu_{12}\mu_{21}} & \frac{R_{22}\mu_{12}}{1-\mu_{12}\mu_{21}} & 0\\ \frac{R_{22}\mu_{12}}{1-\mu_{12}\mu_{21}} & \frac{R_{11}}{1-\mu_{12}\mu_{21}} & 0\\ 0 & 0 & H_{12} \end{array}\right)}^{\left(k\right)}{\left(\begin{array}{c} \varepsilon_{x}\\ \varepsilon_{y}\\ \gamma_{xy} \end{array}\right)}^{\left(k\right)}-{\left(\begin{array}{ccc} \frac{E_{22}}{1-\nu_{12}\nu_{21}} & \frac{E_{22}\nu_{12}}{1-\nu_{12}\nu_{21}} & 0\\ \frac{E_{22}\nu_{12}}{1-\nu_{12}\nu_{21}} & \frac{E_{11}}{1-\nu_{12}\nu_{21}} & 0\\ 0 & 0 & G_{12} \end{array}\right)}^{\left(k\right)}{\left(\begin{array}{c} \alpha_{22}\\ \alpha_{11}\\ 0 \end{array}\right)}^{\left(k\right)}\Delta T+{\left(\begin{array}{c} 0\\ \sigma_{r}V_{s}\\ 0 \end{array}\right)}^{\left(k\right)}$$

where subscripts ‘11’ and ‘22’ indicate the x and y directions, respectively. The SMA composite laminate’s Young’s modulus, shear modulus, and Poisson ratio are represented as R, H and μ, respectively. Moreover, the Young’s modulus, shear modulus and Poisson’s ratio of composite laminates without SMAs are represented as E, G and ν, respectively. The terms $\sigma_{r}$ and $V_{s}$ indicate the recovery stress and the SMA volume fraction. Other equations and terms in Equations (13) and (14) are completed in Appendix A.

The elasticity equation of gradient composite laminates that are reinforced by nitinol/graphite can be expressed as [8]:

(15) $$\left(\begin{array}{c} \left[N\right]\\ \left[M\right] \end{array}\right)=\left(\begin{array}{cc} \left[A\right] & \left[B\right]\\ \left[B\right] & \left[D\right] \end{array}\right)\left(\begin{array}{c} \left[\varepsilon^{0}\right]\\ \left[\Gamma \right] \end{array}\right)-\left(\begin{array}{c} \left[N_{\Delta T}\right]\\ \left[M_{\Delta T}\right] \end{array}\right)+\left(\begin{array}{c} \left[N_{r}\right]\\ \left[M_{r}\right] \end{array}\right)$$

where the extensional rigidity [A], coupling rigidity [B], and bending rigidity [D] of the laminates are defined as:

(16) $$\left(A_{ij},B_{ij},D_{ij}\right)=\int_{-h/2}^{h/2}{\tilde{Q}}_{ij}\left(1,z,z^{2}\right)dz$$

The coupling stiffness matrix [B] is identically zero for symmetric laminates. The incremental recovery stress resultant $\left[N_{r}\right]$ depends on the temperature, pre-strain $\varepsilon_{r}$, and SMA fiber volume fraction $V_{s}$. By utilizing the differential equation of the free vibration of the composite plate [11], the boundary conditions for the simply supported edges of fiber orthogonal laminates are defined as:

(17) $$\{\begin{array}{c} u\left(x,y,t\right)=\sum_{m=1}^{\infty }\sum_{n=1}^{\infty }U_{mn}\cos\frac{m\pi x}{a}\sin\frac{n\pi y}{b}e^{j\omega t}\\ v\left(x,y,t\right)=\sum_{m=1}^{\infty }\sum_{n=1}^{\infty }V_{mn}\sin\frac{m\pi x}{a}\cos\frac{n\pi y}{b}e^{j\omega t}\\ w\left(x,y,t\right)=\sum_{m=1}^{\infty }\sum_{n=1}^{\infty }W_{mn}\sin\frac{m\pi x}{a}\sin\frac{n\pi y}{b}e^{j\omega t} \end{array}$$

The eigenvalue equation is as follows:

(18) $$\left(\begin{array}{ccc} L_{11} & L_{12} & 0\\ L_{12} & L_{22} & 0\\ 0 & 0 & L_{33} \end{array}\right)\left(\begin{array}{c} U_{mn}\\ V_{mn}\\ W_{mn} \end{array}\right)=\left(\begin{array}{c} 0\\ 0\\ 0 \end{array}\right)$$

where:

(19) $$L_{11}=-A_{11}{\left(\frac{m\pi }{a}\right)}^{2}-A_{66}{\left(\frac{n\pi }{b}\right)}^{2}$$

(20) $$L_{12}=-\left(A_{12}+A_{66}\right)\left(\frac{m\pi }{a}\right)\left(\frac{n\pi }{b}\right)$$

(21) $$L_{22}=-A_{66}{\left(\frac{m\pi }{a}\right)}^{2}-A_{11}{\left(\frac{n\pi }{b}\right)}^{2}$$

(22) $$L_{33}=D_{11}\left[{\left(\frac{m\pi }{a}\right)}^{4}+{\left(\frac{n\pi }{b}\right)}^{4}\right]+\left(2D_{12}+4D_{66}\right){\left(\frac{m\pi }{a}\right)}^{2}{\left(\frac{n\pi }{b}\right)}^{2}-\left(N_{\Delta Tx}-N_{rx}\right){\left(\frac{m\pi }{a}\right)}^{2}-\left(N_{\Delta Ty}-N_{ry}\right){\left(\frac{n\pi }{b}\right)}^{2}-\rho \omega^{2}$$

The natural frequency of the two-phase fiber composite laminate can be solved by the eigenvalue equation.

## 3. Results and Discussion

### 3.1. Materials and Verification

**Figure 2:**
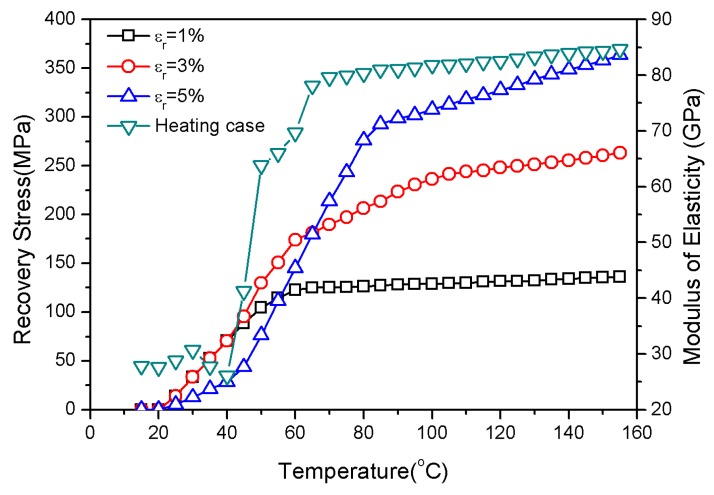
SMA modulus of elasticity and recovery stress vs. temperature.

In the following, SMA-embedded rectangle cross-ply laminates with simply supported edges are studied. Here, composite laminates with lengths of 0.4 m, widths of 0.3 m, and thicknesses of 0.8 mm are considered, as shown in Figure 1. To the constraints of simply supported edges, the composite laminates with two-phase fibers were subjected to small free vibration under thermal loads. The in-plane stress of the laminates was caused by the thermal stress that was produced by the graphite and epoxy resin matrix and the recovery stress that was produced by nitinol. Figure 1 demonstrates that the layout of two-phase fibers were closely enclosed by the epoxy resin. The thermal stress that was generated by the epoxy resin and graphite in the thermal expansion environment acted radially on nitinol along the axis core of the fibers. Similarly, the recovery stress that was produced by the SMA could also react radially on the substrate. The material properties of the fibers and matrix are given in Figure 2 and Table 1 [10].

To facilitate a comparison of research results calculated by Equation (18), the dimensionless natural frequency can be calculated as Equation (23).

(23) $$\varpi =\frac{\omega L_{b}^{2}}{2\pi h}\sqrt{\frac{\rho_{fm}}{E_{22}}}$$

where $E_{22}$ is the transverse elastic modulus of the substrate and $\rho_{fm}$ is the equivalent density of the substrate. The terms ϖ and ω denote the dimensionless natural dimension frequency and natural frequency of the composite laminate, respectively.

**Figure 3:**
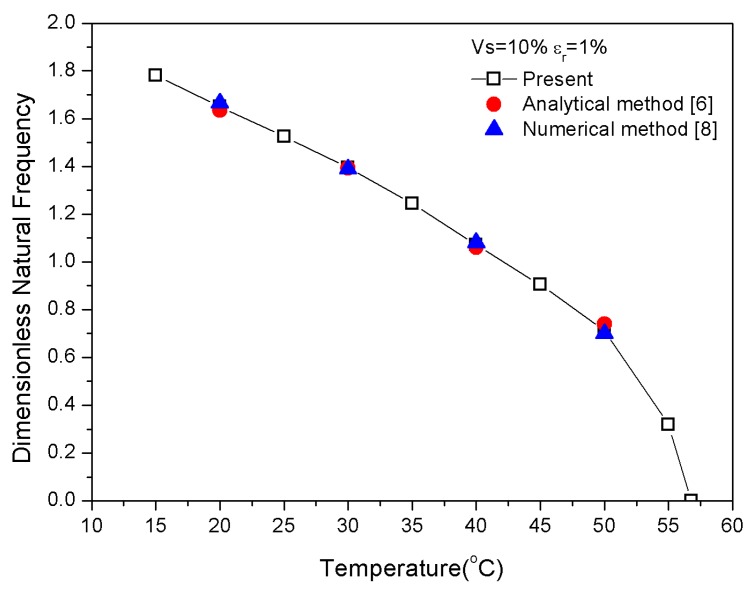
Dimensionless natural frequency vs. temperature.

The physical parameter *P* was taken as zero to verify the correctness of the theoretical model and code that were used in this study according to the material properties in Table 1. Composite laminates with uniform distributions of SMA/graphite were assumed in each layer. Figure 3 verifies the variation in modal frequency with temperature by comparing the results of the present method with analytical [6] and numerical methods (Finite element method) [8]. The comparison of references [5,6,8] in Table 2 certifies the correctness of natural frequencies that were calculated by the present method of different pre-strains and SMA volume fractions taken at 30 °C. Thus, this analytical method was a sound choice for the study.

### 3.2. Influence of Physical Parameter P (Gradient Index) on Frequency

**Figure 4:**
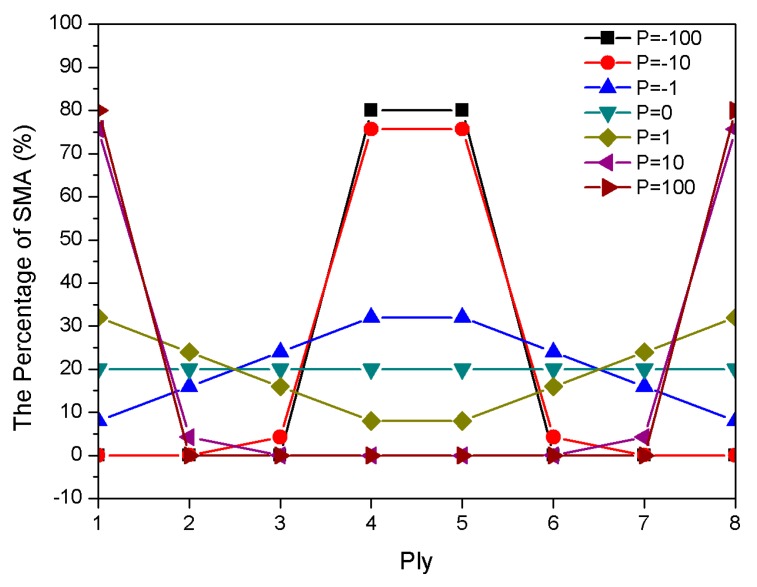
The percentage of SMAs in each ply.

The natural frequency of composite laminates is affected by the distribution of SMAs. The gradient distribution of the SMA was compared to the uniform distribution by using a physical parameter *P* that was exponential and could meet the difference demand of the gradient distribution. Corresponding to a value $p_{0}=0$ means that the SMA content was uniformly laminated, and values of $p_{+}{=10}^{m}\;\left(m=0,1,2\right)$ and $p_{-}{=-10}^{n}\;\left(n=0,1,2\right)$ expressed positive and negative gradients distribution of SMA content variation, respectively, as shown in Figure 4. The calculation of dimensionless natural frequency is based on the assumption that the volume fraction of the shape memory alloy is 20%. The modulus of elasticity and pre-strain that were used in the vibration analysis included the heating case and $\varepsilon_{r}$ = 5%.

**Figure 5:**
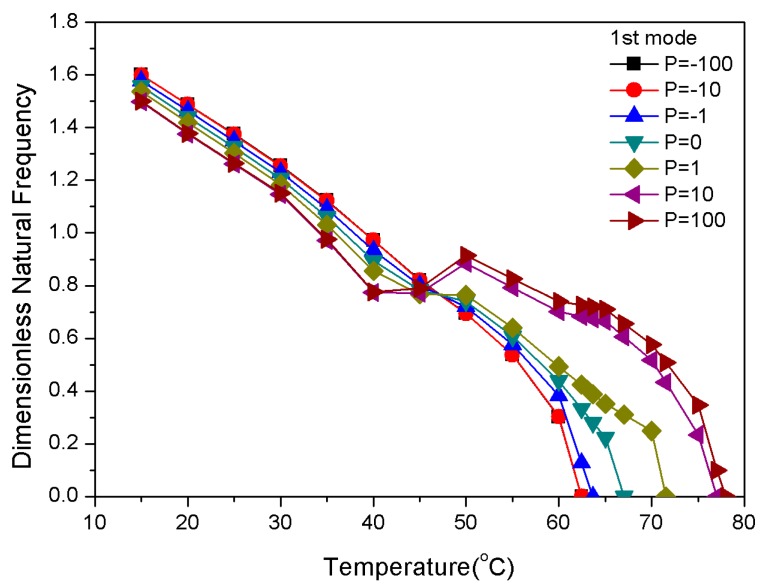
Dimensionless natural frequencies vs. temperature with variations of the physical parameter (P) value.

Figure 5 represents the fundamental dimensionless natural frequencies and the critical buckling temperature of the composite laminates with SMA in a uniform and gradient distribution. The natural frequency tended to decrease as the temperature increased because the bending stiffness factor $L_{33}$ of the composite laminates decreased due to the thermal effect of the epoxy resin substrate. However, different physical parameters *P*, along with rising temperature, could lead to significantly different vibration behaviors. The curve of *P* = 0 in Figure 5 shows that its natural frequency decreased with increasing temperature, followed by a final dropping approach zero at 67 °C, which was at the critical point of buckling.

From 15 to 40 °C, the fundamental dimensionless natural frequency of the SMA in the composite laminates with positive gradient distributions was lower than that with a uniform distribution. The SMA was mainly concentrated in the bottom and top layers of the laminate with a positive gradient distribution, with simultaneously less graphite in the bottom and top layers. From Figure 2, it can be seen that the recovery stress was within 100 MPa due to pre-strain from the SMA within the non-twin martensite phase. The thermal stress that was generated by the substrate played a leading role in the internal forces of the laminate. Therefore, versus the SMA’s uniform distribution laminates, the thermal stress with the SMA had a positive gradient distribution that decreased the natural frequency. The transformation of the martensite phase to the austenite phase promoted an increased recovery stress at temperatures greater than 40 °C. This suggests that the recovery stress led to an increase in the in-plane force, which resulted in a rise in the natural frequency. With continued heating, the martensite phase was completely transformed into the austenite phase, so the recovery stress tended to be relatively stable. The natural frequency decreased to nearly zero due to the continuous decrease in the laminate in-plane forces that was caused by a continuous increase in the thermal stress. The SMA positive gradient distribution could increase the natural frequency and delay the thermal buckling temperature.

When the temperature was 15–40 °C, the influence of the thermal stress that was generated by graphite was dominant because of the lower SMA contents in the bottom and top layers of the composite laminates with a negative gradient distribution of the SMA. The natural frequency of the laminated plates was higher than those of the SMA with a uniform distribution and the SMA with a positive gradient distribution. When the temperature was higher than 40 °C, most of the SMAs were concentrated in the middle layers, and the recovery stress of the SMAs could not be effectively exerted. The natural frequency rapidly decreased as the temperature continued to rise. The critical buckling temperature at which the SMA laminates with a negative gradient distribution was lower than that with a uniform and positive gradient distribution. 

**Figure 6:**
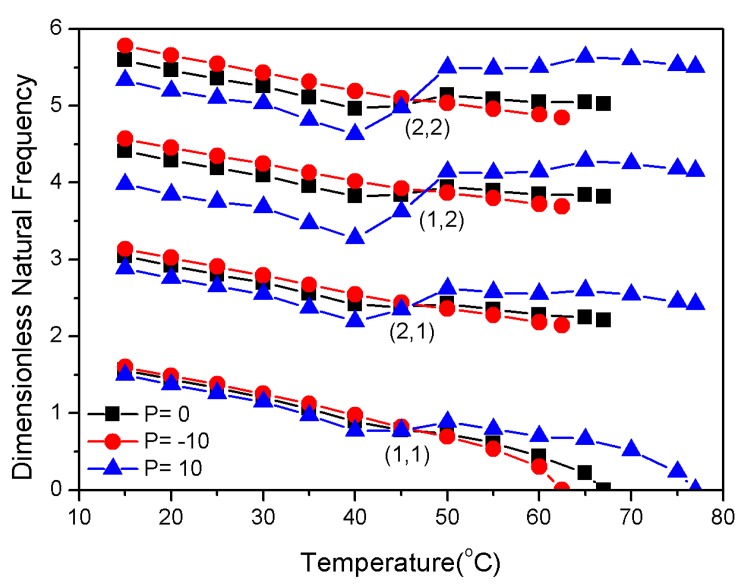
The lowest four order dimensionless natural frequencies.

Table 3 shows the values of modal frequencies and critical buckling temperatures at 30, 45, and 60 °C. As the P value increased, the natural frequency decreased at 30 °C and increased at 60 °C. In addition, there was a positive correlation between the P value and the critical buckling temperature. The critical buckling temperature and the lowest four order dimensionless natural frequency of the composite laminates are shown in Table 3 and Figure 6.

Figure 6 shows that under the same SMA distribution, the lowest four order natural frequencies had the same general trend with temperature. For the uniform and negative gradient distribution of the SMA in the laminates, the natural frequency declined with increasing temperature. However, the positive gradient distribution of the SMA had an inflection point at about 40 °C due to the effect of the recovery stress. The increase recovery stress dominated in a certain temperature range after that; the lowest fourth natural frequency was increased. In addition, there was a temperature between 40 and 50 °C at which the gradient distribution did not affect the modal frequency, i.e., a temperature at which the SMA distribution did not affect the composite.

### 3.3. Influence of Pre-Strain Value on Frequency

Figure 2 shows that the recovery stress varied with temperature at high nonlinearity. The pre-strain caused less recovery stress in the low temperature region. However, the recovery stress increased rapidly with the increasing temperature. Moreover, the initial pre-strain determined the increase of the recovery stress. The recovery stress with a pre-strain of 1% first stabilized during the heating process. However, the recovery stress that was caused by temperature remained relatively stable when the temperature was higher than 100 °C; the recovery stress of 1% pre-strain was less than those of the 3% and 5% pre-strains.

**Figure 7:**
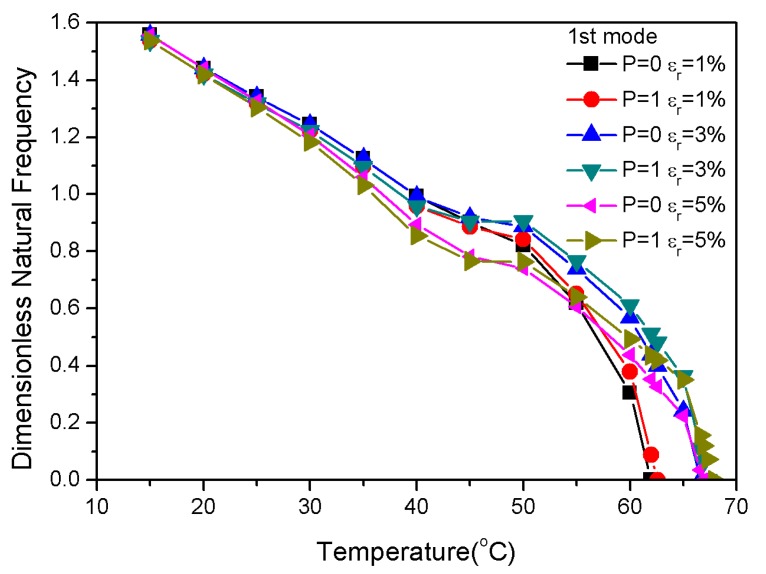
Dimensionless natural frequencies vs. temperature with variations of pre-strain.

The variations of the fundamental frequencies in the pre-strains of 1%, 3% and 5% is shown in Figure 7. Different pre-strains had a slight effect on the natural frequency below 40 °C. The natural frequency decreased with increasing temperature; the critical buckling temperature of 3% pre-strain and 5% pre-strain was higher than that of 1% pre-strain. Figure 7 demonstrates that the critical buckling temperature of P = 1 was higher than P = 0 in the same pre-strain; thus, it is clear that the pre-strain and *p*-values affected the natural frequency and critical buckling temperature. In addition, the recovery stress that was generated by different pre-strains varied greatly above 70 °C; however, the critical buckling temperatures of the fundamental frequency were all below 70 °C, indicating that the large modulus of the graphite in the substrate had a significant effect on natural frequency and buckling temperature. Therefore, the critical buckling temperature of the composite laminates could be tuned via the graphite volume fraction.

### 3.4. Influence of Component Volume Fraction of Materials on Frequency

**Figure 8:**
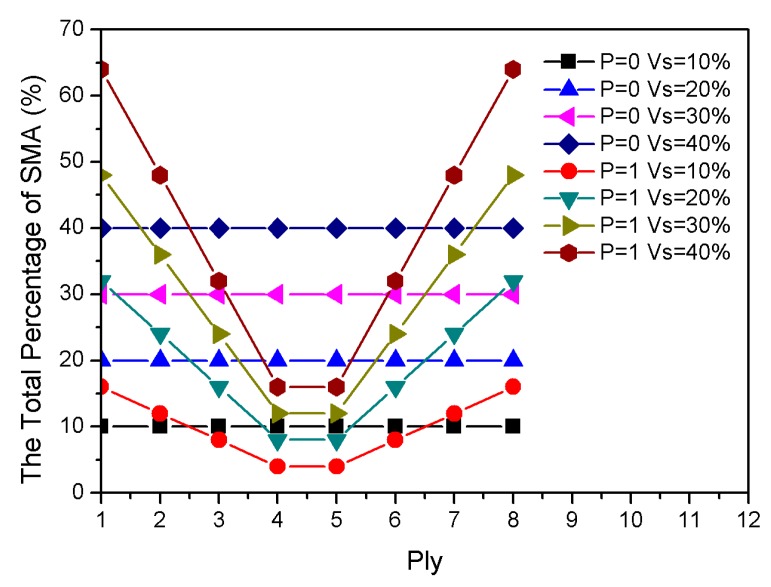
The total percentage of the SMA in each ply.

**Figure 9:**
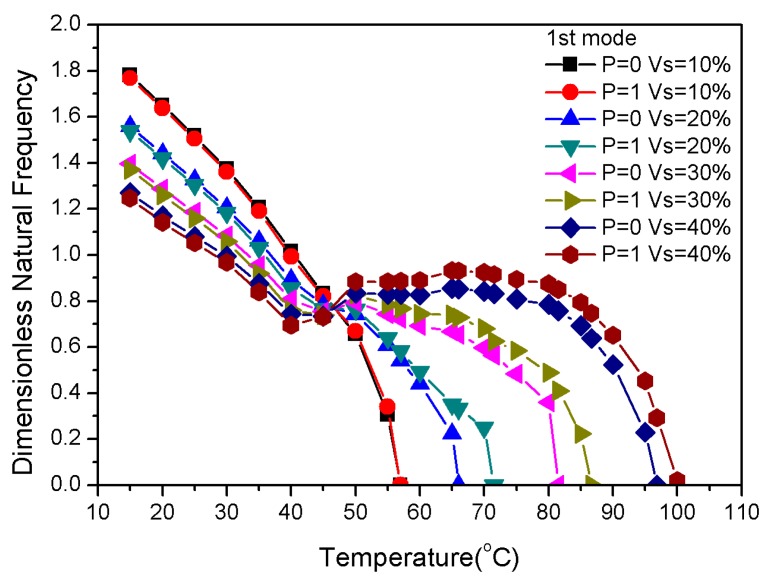
Dimensionless natural frequencies vs. temperature with variations of the total SMA content.

Figure 8 shows the total percentage of the SMA in each ply. Figure 9 displays the influence of the total SMA volume fraction on the composite laminates. Natural frequencies with respect to the variation of the total SMA volume fraction had a frequency intersection at 47 °C. This means that total SMA volume fraction, which had no significant effect on natural frequencies at 47 °C, changed the gradient distribution and the proportion of material content. Additionally, the critical buckling temperature of P = 1 was higher than that of P = 0 when the total content of SMA was constant. More importantly, the total volume fraction of the SMA was directly proportional to the critical buckling temperature. The total SMA volume fraction in the laminate was also directly proportional to the natural frequency when the temperature was higher than 47 °C.

**Figure 10:**
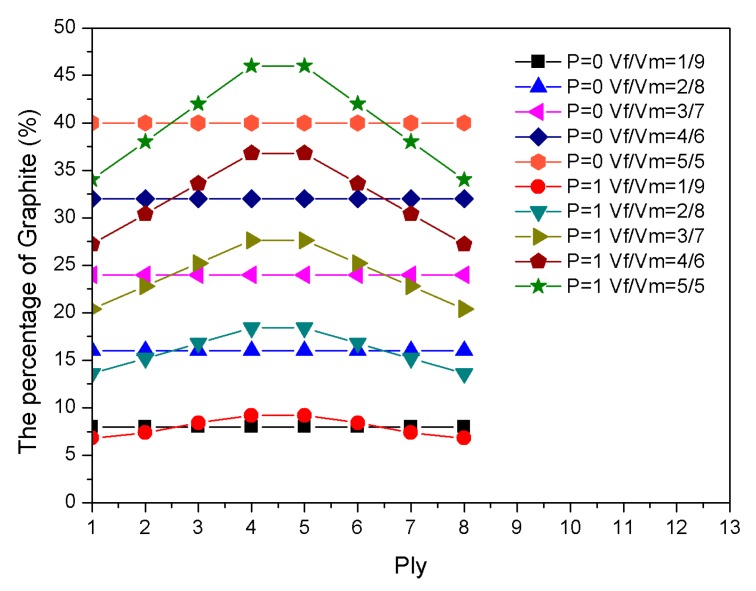
The percentage of graphite in each ply.

**Figure 11:**
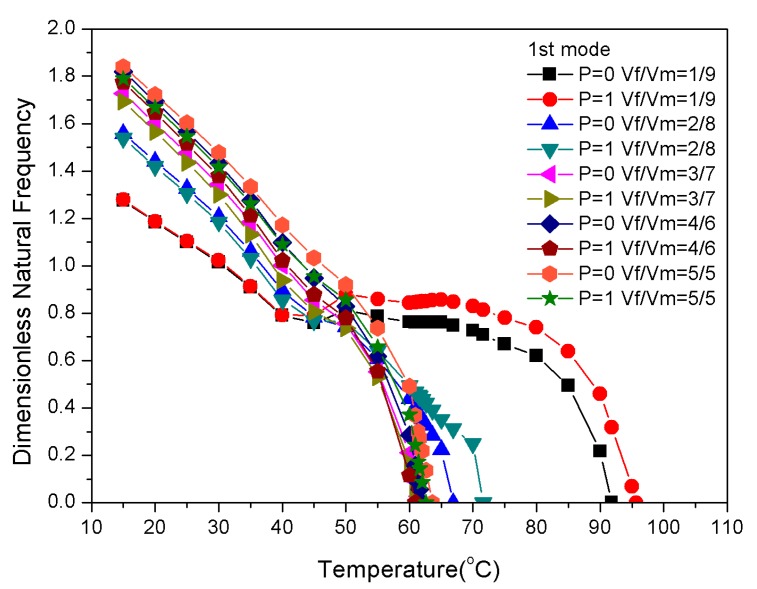
Dimensionless natural frequencies vs. temperature with variations of the substrate ratio.

For the composite laminates that were embedded with two-phase fibers, the graphite content affected the thermal stress, while the shape memory alloy mainly affected the recovery stress of the laminate due to the pre-strain factor. The ratio of the SMA volume fraction to the graphite volume fraction revealed the effect of the recovery and thermal stresses on the natural frequency of the laminate. Figure 10 depicts the percentage of graphite in each ply. Figure 11 highlights the effect of graphite volume fraction on the natural frequencies of P = 0 and P = 1 during the heating process when the SMA volume fraction was constant at 20%. The volume fraction ratio design of the graphite and epoxy resin and the buckling temperature are shown in Table 4.

Figure 11 and Table 4 indicate that since the graphite volume fraction changed from 8% (V_f_/V_m_=1/9) to 16% (V_f_/V_m_=2/8), the critical buckling temperatures for P = 0 and P = 1 changed from 91.8 and 95.7 °C to 66.9 and 71.6 °C, respectively. This was because of the thermal stress that was generated by the large modulus of graphite, which determined the natural frequency reduction process of the laminate. This also determined the critical buckling temperature of the laminate. At V_f_/V_m_=1/9, the critical buckling temperature increased from 91.8 to 95.7 °C when the distribution changed from P = 0 to P = 1. These results prove again the large modulus of graphite has a significant effect on natural frequency.

### 3.5. Influence of Geometrical Properties on Frequency

**Figure 12:**
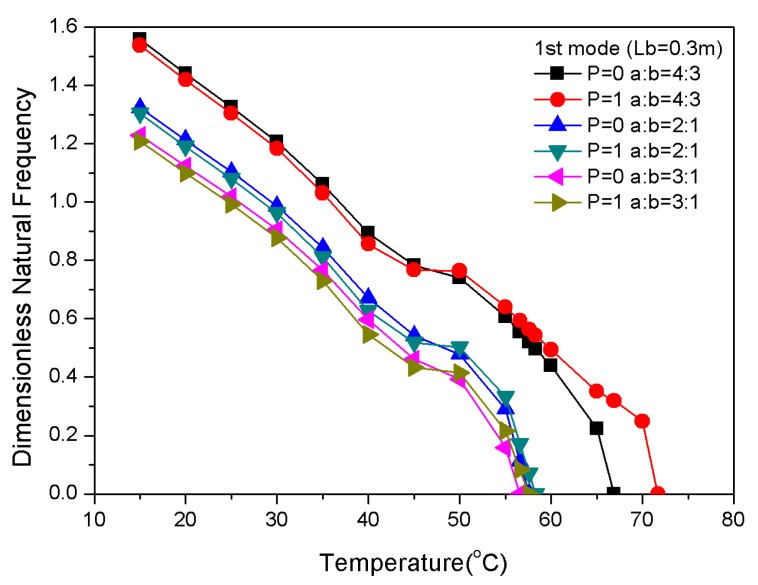
Dimensionless natural frequencies vs. temperature with variations of length.

**Figure 13:**
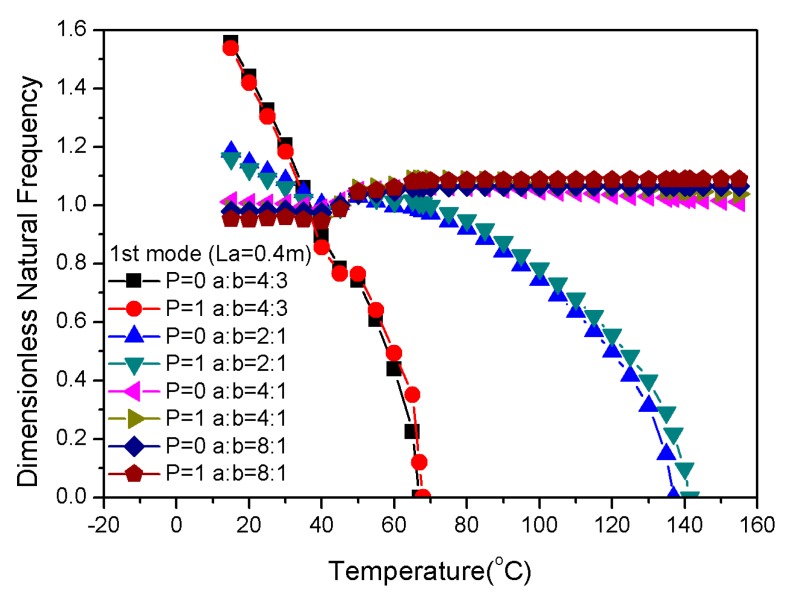
Dimensionless natural frequencies vs. temperature with variations of width.

The structural dimensions of composite laminates with embedded two-phase fibers can be tuned by varying the length, width, and thickness. The observed increase in length for a constant width appeared to increase the board area and decrease the stiffness of the composite laminates (Figure 12). However, at a certain width, the natural frequency gradually decreased with the increasing temperature, and the critical buckling temperature of the positive gradient distribution was higher than the uniform distribution. Figure 13 shows that when the length of the plate was constant, the stiffness increased with decreasing width; thus, the laminate became like a beam, and the natural frequency increased.

**Figure 14:**
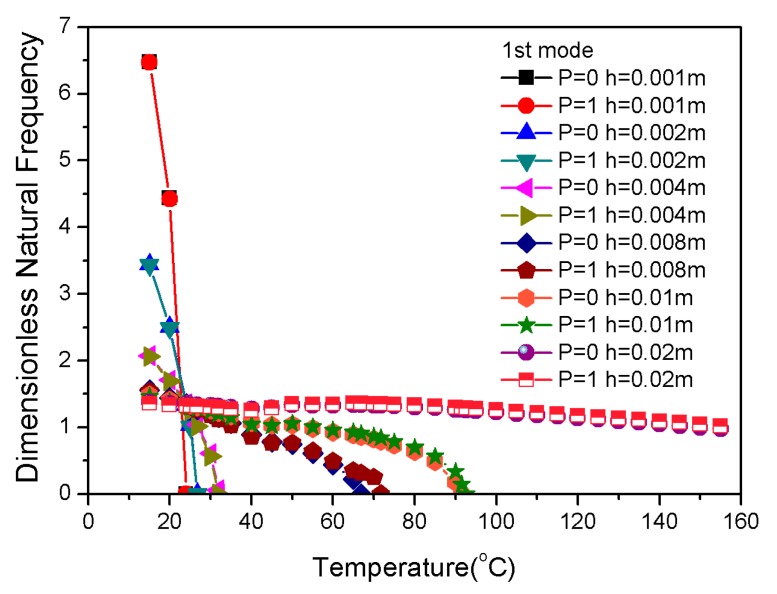
Dimensionless natural frequencies vs. temperature with varying thicknesses.

Another approach that can enhance the stiffness is to increase the thickness. The result of natural frequencies in thickness variations of the laminate is shown in Figure 14. The combination of a positive SMA gradient distribution with increased thickness improved the natural frequency and critical buckling temperature.

## 4. Conclusions

The modal performance of an orthogonal gradient laminate that was embedded with a two-phase fiber with SMA was studied by analytically solving eigenvalue equations. These results suggest the following:

(1) The natural frequency and critical buckling temperature of positive gradient distribution in SMA fibers in composite laminates are higher than those of uniform and negative gradient distributions. Therefore, the positive gradient distribution of SMA fibers is much better for embedding and improves the efficiency of pre-strain to produce recovery stress.
(2) The large modulus of elasticity of graphite affects thermal stress, while the pre-strain and distribution of nitinol affect in-plane recovery stress. The natural frequency and critical buckling temperature of composite laminates can be effectively adjusted by controlling the internal force of the composite laminate with the proportion and content of graphite and SMAs.
(3) When the SMA volume fraction is constant, the reduction of the width or the increase of the thickness of composite laminates can effectively increase the natural frequency and the critical buckling temperature.

## Figures and Tables

**Table 1:** Material properties of the shape memory alloy (SMA), graphite, and epoxy matrix.

SMA		Graphite		Epoxy	
$E_{s}$ (GPa)	From Figure 2	$E_{f}$ (GPa)	275.6	$E_{m}$ (GPa)	3.43
$\sigma_{r}$ (MPa)	From Figure 2	$G_{f}$ (GPa)	114.8	$G_{m}$ (GPa)	1.27
$v_{s}$	0.3	$v_{f}$	0.2	$v_{m}$	0.35
$\rho_{s}$ (kg·m^3^)	6450	$\rho_{f}$ (kg·m^3^)	1900	$\rho_{m}$ (kg·m^3^)	1250
$\alpha_{s}$ (1/°C)	10.26 × 10^−6^	$\alpha_{f}$ (1/°C)	24.40 × 10^−6^	$\alpha_{m}$ (1/°C)	64.80 × 10^−6^

**Table 2:** Comparison of dimensionless natural frequencies.

		Dimensionless Natural Frequency (*ω*)			
T = 30 °C		Present	Malekzadeh [5]	Mahabadi [6]	Park [8]
$\varepsilon_{r}$ = 1%	Vs = 5%	1.497	1.496	1.488	-
	Vs = 10%	1.395	1.393	1.392	1.389
	Vs = 15%	1.312	-	1.307	1.303
Vs = 15%	$\varepsilon_{r}$ = 3%	1.310	1.308	1.303	1.300
	$\varepsilon_{r}$ = 5%	1.283	-	1.279	-

**Table 3:** Natural frequency and critical buckling temperatures.

P	*ϖ* (T = 30 °C)	*ϖ* (T = 45 °C)	*ϖ* (T = 60 °C)	T_cr_ (°C)
−100	1.255	0.820	0.302	62.5
−10	1.254	0.820	0.303	62.5
−1	1.230	0.802	0.383	63.7
0	1.206	0.783	0.439	67.0
1	1.184	0.767	0.493	71.5
10	1.145	0.773	0.702	77.0
100	1.151	0.789	0.741	78.0

**Table 4:** Critical temperatures.

V_f_/V_m_	P = 0 T_cr_ (°C)	P = 1 T_cr_ (°C)
1/9	91.8	95.7
2/8	66.9	71.6
3/7	61.6	61.4
4/6	62.0	60.9
5/5	63.6	62.6

## Appendix A

The schematic of the unit structure shows that the epoxy resin and the graphite fiber were used as the substrate; the composite structure that was formed by the SMA fiber and the substrate satisfied the mixing rule with the composite material. The formulas that were used for the calculation of these effective engineering constants are based on the rule of mixture [5,6].

(A1) $$E_{11}=E_{m}V_{m}+E_{f}V_{f}$$

(A2) $$E_{22}=\frac{E_{m}E_{f}}{E_{m}V_{f}+E_{f}V_{m}}$$

(A3) $$\nu_{12}=\nu_{m}V_{m}+\nu_{f}V_{f}$$

(A4) $$\nu_{21}=\nu_{12}E_{22}/E_{11}$$

(A5) $$G_{12}=\frac{G_{m}G_{f}}{G_{m}V_{f}+G_{f}V_{m}}$$

(A6) $$\alpha_{11}=\frac{E_{11}\alpha_{m}V_{m}+E_{f}\alpha_{f}V_{f}}{E_{11}}$$

(A7) $$\alpha_{22}=\alpha_{m}V_{m}+\alpha_{f}V_{f}$$

(A8) $$R_{11}=E_{11}\left(1-V_{s}\right)+E_{s}V_{s}$$

(A9) $$R_{22}=\frac{E_{s}E_{11}}{E_{s}\left(1-V_{s}\right)+E_{11}V_{s}}$$

(A10) $$\mu_{12}=\nu_{12}\left(1-V_{s}\right)+\nu_{s}V_{s}$$

(A11) $$\mu_{21}=\mu_{12}R_{22}/R_{11}$$

(A12) $$H_{12}=\frac{2G_{12}E_{s}}{2G_{12}V_{s}\left(1+\nu_{s}\right)+E_{s}\left(1-V_{s}\right)}$$

(A13) $$\rho_{fm}=\rho_{f}V_{f}+\rho_{m}V_{m}$$

(A14) $$\rho =\rho_{fm}\left(1-V_{s}\right)+\rho_{s}V_{s}$$

where subscript ‘s,’ ‘f,’ and ‘m’ denote the SMA fibers, the graphite fiber, and the epoxy resin, respectively.
